# A Scoping Review of Bystander-Based Sexual Violence Prevention Training for College Students in Fraternities and Sororities

**DOI:** 10.3390/ijerph21060797

**Published:** 2024-06-19

**Authors:** Caterina DeFazio, Samantha I. Moyers-Kinsella, Elizabeth A. Claydon, Michelle D. Hand, Christa Lilly, Keith J. Zullig, Danielle M. Davidov

**Affiliations:** 1 Department of Social and Behavioral Health Sciences, West Virginia University, Morgantown, WV 26506, USA; 2 Center for Active WV, West Virginia University, Morgantown, WV 26506, USA; 3 Department of Social Work, George Mason University, Fairfax, VA 22030, USA; 4 Department of Epidemiology and Biostatistics, West Virginia University, Morgantown, WV 26506, USA

**Keywords:** bystander intervention training, sexual assault prevention, Greek life, scoping review, fraternity, sorority, Title IX

## Abstract

Bystander-based sexual violence (SV) prevention trainings are offered on college campuses across the United States to meet federal Title IX requirements, as they have proven to be an effective strategy for violence prevention. Greek-affiliated students (fraternity and sorority members) are at a higher risk of sexual assault than their peers; however, few trainings consider the specific needs of this population, and program adaptations for this high-risk group may be needed. This scoping review identifies and describes the bystander trainings delivered to Greek-affiliated students in the US and Canada. An eight-database search was conducted following PRISMA-ScR guidelines. The review identified 81 unique sources, with 18 meeting the inclusion criteria. Eleven specific training programs were identified, encompassing qualitative, quantitative, and mixed-method studies. The thematic analysis revealed best practices, including the importance of peer leaders, interactive sessions, and tailored content to Greek culture, as well as barriers such as a lack of engagement and an inadequate session length. The review underscores the need for tailored interventions to effectively address the unique cultural characteristics and high-risk nature of Greek-affiliated students. These findings provide valuable insights for improving the design and implementation of bystander interventions to enhance their efficacy in preventing sexual violence within this population.

## 1. Introduction

Sexual assault (SA) is a significant and concerning public health issue that is associated with numerous physical and psychological health consequences [1]. Physically, SA victims and survivors may contract sexually transmitted infections (STI), have an unwanted pregnancy, or experience genital and/or non-genital trauma [1]. Psychologically, a wide range of short- and long-term mental health impacts can affect victims (e.g., posttraumatic stress disorder (PTSD), substance use disorder (SUD), depression, anxiety, obsessive-compulsive disorder (OCD), and eating disorders) [2,3,4].

SA on college campuses in the United States (US) is prevalent and was labeled an epidemic by the Obama Administration in 2014 [5]. Estimates demonstrate that approximately 31% of college-attending women and 10% of college-attending men (18–24 years of age) are sexually assaulted during their collegiate careers [6] Evidence also demonstrates that college-attending men are more likely (14.5%) than women (3.8%) to be perpetrators of SA. Given that rates of SA rely primarily on self-report, it is recognized that the current incidence and prevalence data are likely underestimates. For example, only 20% of women who have been sexually assaulted report the assault [7]. Canadian campuses have similar estimates of SA [8]; however, most of the existing literature comes from the US due to legislative mandates that exist in the US but not in Canada [9]. 

Between 2011 and 2016, increased attention and legislative changes resulted in college campuses re-evaluating how they address SA; many have added prevention and intervention resources and revised how cases of SA are handled by universities [10]. Additionally, in 2013, the Cleary Act was revised, specifically mandating that campuses receiving Title IX funds provide all students with education about SA and SA prevention, including the implementation of trainings or programs that contain bystander intervention elements [11]. As a result of this directive, various bystander intervention trainings have been developed and evaluated. In the bystander approach to violence prevention, students typically learn the warning signs of SA (e.g., slipping drugs into a drink, verbal abuse, physical signs of abuse), as well as direct and indirect intervention techniques (e.g., direct intervention, distraction, delegation) to prevent or help someone they perceive to be at risk of SA [12]. This bystander approach to SA prevention aims to shift norms to prevent and reduce SA by enhancing students’ collective efficacy by making them feel accountable for one another. Popular bystander-based training programs include *Green Dot*, *Bringing in the Bystander*, and *Step Up* [13,14,15].

Many bystander intervention programs employ a one-size-fits-all approach to prevention, aiming to increase awareness, knowledge, and skills among the general student body [16,17]. It is common for programs to deliver speeches to large groups or students in classrooms, typically to first and second-year college students [15]. Select groups on campus that may be influential in SA prevention efforts, or that may be at risk of SA perpetration and/or victimization, such as athletes and students involved in fraternity and sorority life, are sometimes specifically targeted to receive bystander-based violence prevention programming; however, the extent to which existing and/or evidence-based programs may need to be adapted to the norms, values, and other cultural characteristics of these student communities is unknown. 

Multiple studies have demonstrated that fraternity and sorority members (hereafter referred to as Greek-affiliated students) are at the greatest risk of SA victimization and perpetration on college campuses [18,19,20]. Notably, women in sororities are 74% more likely to experience SA than those not involved with sororities [21]. These increased rates of SA have been attributed to the characteristics associated with fraternity and sorority culture, such as high rates of binge drinking, sexually hostile peer culture, and heteronormative gender norms [22,23,24,25,26]. The literature demonstrates that men in fraternities have more undesirable attitudes towards sexual violence and sexual consent [27] and are more likely than their non-Greek-affiliated counterparts to report committing sexual assault (8% vs. 1% [22]). Students in fraternities have also reported more negative attitudes toward bystander action (i.e., speaking up or intervening) in SA situations, and some evidence even suggests that joining a Greek organization may exacerbate these attitudes and behaviors over time [28]. Thus, it is possible that Greek-affiliated college students need tailored SA prevention content and messaging that acknowledge their distinct norms and experiences in order to be effective. The literature suggests that tailoring bystander intervention trainings explicitly for Greek-affiliated students might aid in reducing the rates of SA, but there is a dearth of literature on whether and how campuses are implementing these [14,29,30,31]. Further, it is unknown which existing bystander-based SA prevention programs are being implemented with students involved in Greek Life. It is possible that programs developed and/or adapted for a specific campus (e.g., GUARD [32], FratMANners and SISSTER [33], and One ACT for Greeks at the University of North Carolina [34]) have not been widely reported in the scientific literature or evaluated for efficacy. 

Beyond concerns for student safety and well-being, college campuses have a vested interest in preventing and reducing SA via bystander-based education. If parents, students, and alumni doubt the university’s commitment and ability to combat violence, it may undermine its perceived safety [35]. Further, high rates of SA are associated with declines in academic performance and lower graduation rates, which can negatively affect the reputation of institutions of higher education [36]. This may also directly affect institutional funding, as high-profile cases of SA have been shown to directly affect monetary donations and funding related to enrollment, resulting in significant legislative fines and university investigations [35].

Due to the emergence of bystander interventions, which have demonstrated efficacy in reducing SA [13], it is important to understand how these trainings can be developed and adapted to groups at the highest risk of SA victimization and perpetration. Thus, the main objective of this study was to conduct a scoping review of the literature to examine existing bystander intervention trainings delivered to Greek-affiliated college students in the US and Canada. In this study, we provide detailed information on the program characteristics as well as study design features, including commonly used scales to measure training efficacy. The other objectives of this scoping review include (a) identifying barriers and best practices to the implementation of these interventions and (b) identifying gaps in the literature to further improve bystander intervention trainings for this high-risk population. These findings have the potential to inform violence prevention efforts at academic institutions where SA is pervasive [18,19,37].

## 2. Methods

### 2.1. Study Design

This study used a scoping review design. Scoping reviews are time-efficient literature reviews that are conducted to map the current literature on a given topic and include aspects such as the volume of primary publications and their nature and characteristics [38]. This review method is used when there is a dearth of literature regarding a more niche and sensitive subject matter [39]. Due to the limited literature on bystander intervention trainings that target Greek-affiliated students, a scoping review is appropriate for exploring this topic. Unlike systematic reviews, scoping reviews do not include a meta-analysis or a statistical summary of the results of the reviewed literature, in consideration of the complex nature of and limited research on the topics reviewed, which often involve more qualitative than quantitative research. Once the research questions were established, search terms were developed in collaboration with the other authors and updated throughout the search process to ensure the search was as extensive as possible [40]. Articles were identified using PRISMA Extension for Scoping Reviews (PRISMA-ScR). Then, critical appraisal was conducted to assess the quality of the articles included in this scoping review [41]. The review protocol was registered in Open Science Framework and can be found at https://doi.org/10.17605/OSF.IO/V8X7H (accessed on 26 May 2024).

### 2.2. Search Strategy

Eight electronic databases (EbscoHost, PubMed, Web of Science, PsychINFO, Google Scholar, Scopus, ProQuest Dissertations and Theses, and Academic Search Complete) were searched using the following key search terms: “Greek” OR “Greek Life” OR “Greek organization” OR “Greek society” OR “fraternity” OR “frat” OR “sorority” OR “Panhellenic” OR “IFC” AND “Bystander Intervention” OR “Bystander program” OR “Sexual Violence Intervention” “Sexual Violence Prevention” OR “Sexual Assault Intervention” OR “Sexual Assault Prevention”. Once studies were identified, their reference lists were reviewed for other potential studies for inclusion. A detailed audit trail [42] that included the thematic outcome columns was kept in Microsoft Excel, as well as a section for the interpretations and methodologies used in all searches, data extraction, and synthesis to increase this study’s transferability, confirmability, and dependability [43]. 

### 2.3. Inclusion Criteria

**Figure 1:**
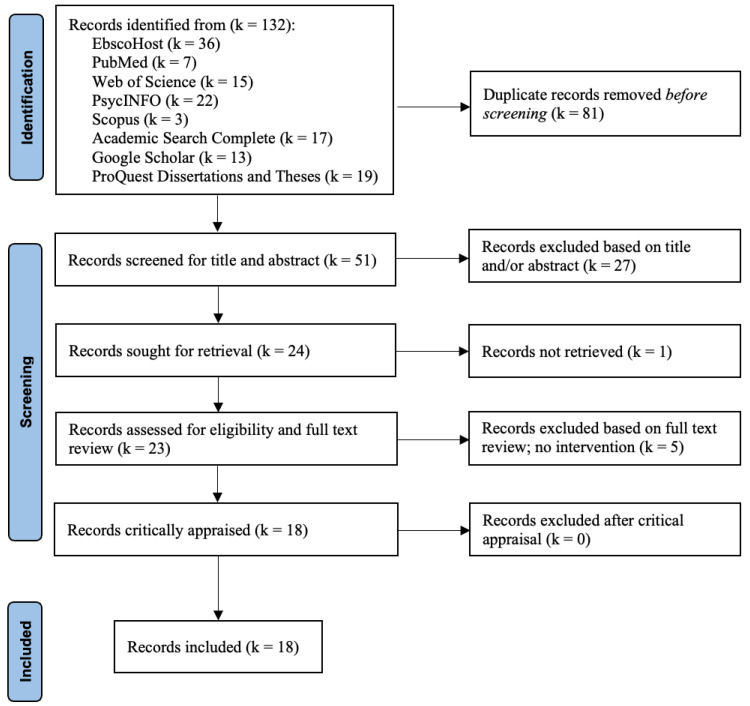
PRISMA Flow Diagram.

This scoping review included studies that examined bystander intervention trainings with a focus on sexual assault that have been implemented for Greek-affiliated students (Figure 1). Studies were eligible for inclusion if they were (1) qualitative, quantitative, or mixed-methods research; (2) peer-reviewed or grey literature (i.e., reports, conference presentations, or theses and dissertations); (3) primary literature (i.e., not a secondary analysis of existing data); (4) published in the English language; (5) published after 2004; (6) focused on one or more bystander intervention trainings implemented for Greek-affiliated college students on college campuses (7) to prevent SA; and (8) conducted in the United States and Canada. 

### 2.4. Study Design

Initially, titles were assessed, and those not meeting the eligibility criteria were excluded. If it needed to be clarified whether a title met the inclusion criteria, an abstract review was performed. If eligibility was still uncertain, a complete document review was conducted [40]. All documents that met this preliminary review were read in their entirety, and those that did not meet the criteria were logged but excluded from the final review. This study implemented dual coding to decrease data extraction bias and increase the accuracy and consistency [44]. Two authors (CD and SMK) reviewed each piece of literature separately in individual codebooks. Once the authors completed this initial coding phase, they met and compared codes. Any items they disagreed on a result, the result was discussed until a consensus was reached. A third author (DD) was available for consultation if the two coders could not reach a consensus. However, there was no instance in which the coders could not reach a consensus. All coding was completed in Microsoft Excel (Version 16.86).

### 2.5. Quality Appraisal

After the articles were located, an in-depth review using Caldwell and colleagues’ critical appraisal framework was carried out to appraise the quality of each source [45]. This framework helps describe the trustworthiness and authenticity of both qualitative and quantitative research studies. The framework consists of 18 questions (e.g., is the literature review comprehensive and up to date, is the methodology identified and justified, and are the results generalizable/transferable) with a rating scale of 0 to 2 (0 = no, 1 = partly, 2 = yes) for a total of 36 potential points. Once the collected literature was reviewed and points were assessed, they were assigned a quality category: >90% (>32 pts) = high quality, 75–90% (27 pts–32 pts) = moderate quality, and 60–74% (22 pts–26 pts) = low quality. Butler and colleagues (2016) have recommended that a thematic analysis is not conducted on studies with quality <60% (<22 pts); however, because some of the included sources were from gray literature (e.g., conference papers and presentations) that may not have contained enough detail for a thorough quality review, we opted to conduct thematic analysis and abstract the relevant data for all sources meeting the inclusion criteria [46]. 

### 2.6. Data Analysis

The data analysis of this study was primarily qualitative, describing the study characteristics and then conducting thematic synthesis. Thematic synthesis consisted of three steps: (1) coding the text, (2) developing descriptive themes, and (3) generating analytic themes (i.e., thematic analysis). Once the quality appraisal was complete, each piece of literature was read, re-read, and coded [46]. Codes were recorded in Microsoft Excel to organize the concepts between studies. Meaning was assigned to codes to develop descriptive themes, which were then organized into categories. These categories were compared, and similar categories were combined into concepts and then themes. These final themes give an overview of the existing literature, resulting in a synthesized understanding of the phenomenon [46] surrounding bystander intervention trainings in Greek-affiliated students on university campuses.

## 3. Results

During the review, 132 sources were identified, and after duplicates were removed, 81 unique sources remained. After screening titles and abstracts, 23 sources were reviewed, and 18 met the inclusion criteria for this scoping review. Nine (50%) of the included studies were Dissertations or Theses, and seven were published in peer-reviewed journals. There was one conference paper and one conference presentation (See Table 1). 

The quality appraisal scores ranged from 19 to 34 points. Two sources received high-quality scores (33–34 points), and 11 were of moderate quality (27–32 points). Four sources were scored as low quality (23–26), and one received 19 points (see Table 2). 

Two studies utilized a mixed-methods approach, one was purely qualitative, and the remaining 15 were quantitative. Of these, there were two single-group studies, five randomized control studies, and eleven quasi-experimental studies. Ten of the studies included a control group. Four studies did not specify a bystander intervention training by name; of the 14 that did, 11 unique trainings were mentioned. *Bringing in the Bystander* was described in four studies, and *Safe Sisters* was included in two. Other named trainings included *RESPECT*, *SWAT* training (Sexual Wellness Advocacy Team), *SWAT Plus*, *The Men’s Program*, *The Women’s Program*, *Conversations and Pizza (CAP)*, *Men in Violence Prevention (MIVP)*, *Sexpectations: Healthy Relationships and Sexuality for College Students*, *Ten Man and Ten Woman Plan*, and *Title IX Training*. 

All studies were conducted at universities in the United States: six were in the Southern geographic area, three were Midwestern, three were Northeastern, two were Pacific Southwestern, one was Pacific Northwestern, and one was Central. One study did not indicate location. 

Twelve studies focused specifically on Greek-affiliated students (six on sorority members, five on fraternity members, and one on Greek-affiliated undergraduate students). Six studies included the entire undergraduate student body (one on undergraduate women and one on undergraduate men, specifically) but contained separate data/analyses on Greek-affiliated students. Only four studies noted a participation requirement from the university for Greek-affiliated students. 

Fifteen studies described in-person bystander intervention training. Twelve studies reported that the bystander intervention training was completed in one session (55 min to 4 h), one training (unspecified) was implemented over 21 weeks, and the *Ten Man and Ten Woman Plan* included weekly 1 h sessions over nine weeks. *Men in Violence Prevention (MIVP)* consisted of two 1 h sessions, and *Conversations and Pizza (CAP)* comprised six sessions over three months. In two studies, information about the number of sessions was not included. 

Nine of the studies used the Bystander Efficacy Scale to measure the efficacy of bystander training and another nine used the Illinois Rape Myth Acceptance Scale. Other frequently used measures included the Bystander Behavior Scale (k = 4), Bystander Intention to Help Scale (k = 4), Sexual Consent Scale (k = 3), Decisional Balance Scale (k = 2), Rape Empathy Scale (k = 2), Readiness to Change (k = 2), Readiness to Help Scale (k = 2), and the Sexual Experiences Survey (k = 2). Other measures covered topics including alcohol (k = 5), sexual violence knowledge (k = 5), bystander intention (k = 3), bystander behavior (k = 2), sexual health (k = 4), risk (k = 2), attitudes (k = 6), victimization (k = 2), perpetration (k = 3), consent (k = 1), communication (k = 1), sexual history (k = 1), and the assessment of interventions (k = 3). The Marlowe–Crowne Social Desirability Scale was used in two studies. 

Most studies (k = 6) began and ended on the same day. However, the studies’ pre–post test period ranged from 1 day to 27 weeks, with the most common period being one day (k = 6). Other periods were two weeks (k = 1) 4 weeks (k = 2), five weeks (k = 2), six weeks (k = 1), eight weeks (k = 1), nine weeks (k = 1), 12 weeks (k = 2), 12–16 weeks (k = 1), and 27 weeks (k = 1).

All of the quantitative studies reported some significant outcomes as a result of the intervention. Similarly, those studies with qualitative methods reported perceived positive outcomes post-intervention. Of note, Childers (2011) found that Greek affiliation did not affect bystander efficacy, willingness to engage in bystander behavior, and readiness to change scores [48]. Smith (2013) noted that Greek-affiliated students experienced a significant reduction in alcohol risk after receiving the intervention [58]. Additionally, Ortiz and Shafer (2018) found that Greek-affiliated students improved in all outcomes at the six-week and 21-week follow-up period, compared with non-Greek students, whose improvements were only observed after the 21-week follow-up period [61]. The outcomes included attitudes about establishing sexual consent, behavioral control to obtain consent, intentions to ask for consent and stop if consent is rescinded, and understanding what sexual consent and sexual assault are. Table 2 provides details of these findings for each study.

The findings were organized thematically into (1) Best Practices for Implementation and (2) Barriers to Implementation. Best Practices for Implementation refer to the processes and content utilized by those implementing the training(s) that resulted in a positive response or outcome or were perceived to have been responsible for training success. Barriers to implementation are the obstacles or challenges encountered by those implementing the training(s). There was agreement between studies regarding which factors were either a best practice or barrier for the themes of Relatability, Comfort, Engagement, and Content Quality. However, there were some discrepancies regarding the theme of Modality, for example, as whether an online modality was considered a barrier or a best practice varied in the studies. Table 3 provides details of these findings for each study.

### 3.1. Best Practices for Implementation

The thematic analysis revealed several key perceived best practices for implementing bystander intervention trainings with Greek-affiliated students. These best practices are grouped into five main themes: Relatability, Comfort, Engagement, Content Quality, and Modality.

#### 3.1.1. Relatability

Notably, 10 out of 18 sources highlighted the importance of relatability in bystander intervention trainings. Peer leaders serving as training facilitators emerged as the most frequently mentioned best practice (k = 4). Studies indicated that trainings led by individuals within the Greek Life community were better received by participants. Collaboration between researchers and Greek Life organizations also surfaced as a subtheme (k = 3), with studies highlighting the involvement of Greek-affiliated students in the planning and implementation of trainings [49,61], and one mentioned that fraternity participants expressed a desire for training collaboration with sororities [62]. Using real-life scenarios in training content was another repeated subtheme (k = 2), with participants valuing survivor stories [62] and real-life situations [57].

#### 3.1.2. Comfort

Creating a comfortable environment for participants was a crucial aspect of effective training (k = 6). Specifically, studies noted the absence of judgment, the provision of single-gender settings, conducting sessions in naturalistic and informal settings (k = 3), and an emphasis on avoiding scare tactics in favor of action-oriented and helping-focused approaches. Notably, Darlington (2014) observed the importance of tailoring the audience to a single gender, making it more effective for women but less crucial for men [49].

#### 3.1.3. Engagement

Seven sources emphasized the need for bystander intervention trainings to be engaging. This included the importance of students’ interest in the content, using incentives to motivate participation (k = 3), engaging speakers who could effectively convey the message, buy-in from campus stakeholders (e.g., staff and administrators), and incorporating interactive elements to enhance engagement. The use of incentives was noted, and Bluth (2014) found that monetary incentives facilitated individual engagement [29]. Among the studies, three highlighted interactive training as a strength, indicating that participants engaged more effectively with the content [57,61,62]. 

#### 3.1.4. Content Quality

Ensuring that the training’s content was of high quality was also a significant concern in two sources. The findings highlight the use of evidence-based information to support the training’s messages, focusing on environmental or group change and the desire for content that participants easily understood. Darlington (2014) highlighted that an important evidence-based aspect of their training focused on environmental/group change by teaching participants how to support survivors [49]. Additionally, participants in the Pinkerton (2011) study were complimentary of the digestibility of the content, stating that the training was “easy to understand” [57].

#### 3.1.5. Modality

Various modalities for delivering the training were discussed in eight sources. Participants highlighted the effectiveness of in-person sessions (k = 3), online modules, videos as teaching tools, multi-session trainings, single-session trainings, and a requirement for participation as factors that contributed to training success. Nevertheless, it proved to be more effective than mixed-sex groups. Three studies highlighted in-person training as a best practice [51,61,62]. However, Ortiz and Shafer (2018) highlight the valuable role played by online tactics in facilitating conversations and reaching a larger portion of the student population [61]. Bluth (2014) noted that the longer format (2 sessions, 4 h) of the training “Bringing in the Bystander” had higher levels of construct validity compared to the shorter format (1 session, 90 min) used in their study [29]. Conversely, Childers’ (2011) single-session training yielded results similar to those of a multi-session study [48].

### 3.2. Barriers to Implementation

The thematic analysis also revealed several barriers to implementing bystander intervention trainings among Greek-affiliated college students. These barriers are grouped into four main themes encompassing the inverse of the aforementioned best practices: Lack of Relatability, Discomfort, Lack of Engagement, and Modality.

#### 3.2.1. Lack of Relatability

One source identified that a lack of relatability is a barrier to training implementation. Darlington (2014) noted that some peer facilitators were not Greek-affiliated, which may have impacted their relatability [49]. According to Darlington (2014), this dichotomy between the facilitator and the target audience hindered students’ ability to connect with and internalize the training’s message [49].

#### 3.2.2. Discomfort

The theme of discomfort highlighted barriers related to the comfort level of participants in three sources. Subthemes included the perception of male-targeted content, discomfort in large audiences or formal settings, and the potential alienation of participants due to these factors. Pinkerton (2011) received feedback from male participants who perceived the training as sexist and felt that men were unfairly blamed. This study also revealed participants’ desire for the training to be co-ed [57].

#### 3.2.3. Lack of Engagement

Engaging participants in bystander intervention training proved challenging due to several factors (k = 4). These barriers included a lack of incentives to motivate participation, trainings that were not interactive and failed to actively engage students, competition with other trainings that diverted students’ attention, and the need to secure buy-in from various campus stakeholders, suggesting that without support, implementation struggles. The use of incentives was noted by Bluth (2014), finding that monetary incentives facilitated individual engagement; however, incentives for Greek organizations as a whole were lacking [29]. Additionally, Davis and colleagues (2004) emphasized the didactic nature of their training and suggested that increased participant involvement would have been beneficial [50].

#### 3.2.4. Modality

The modality of the training played a significant role in its perceived success or failure (k = 4). Barriers to this theme included short sessions that may not have allowed for adequate topic exploration, long sessions that may have been perceived as burdensome, and one-session trainings that may not have had sufficient depth (k = 2). Pinkerton (2011) noted that participants perceived the length of the sessions as unnecessary, suggesting that a higher frequency but shorter length might be better received [57].

## 4. Discussion

This scoping review provides a comprehensive examination of bystander intervention trainings delivered to Greek-affiliated college students and demonstrates that these trainings can be applied to this population. The identified themes and subthemes offer essential insights into the perceived best practices and barriers associated with the implementation of these trainings. The findings underscore the need for training developers and educators to carefully consider and incorporate these elements, with a keen focus on relatability, comfort, engagement, and modality. By addressing these issues, training designers can significantly improve the efficacy and acceptability of their training initiatives.

The efficacy of bystander interventions facilitated by peer opinion leaders (POLs), has been well documented in the extant literature, signifying their potential to disseminate messaging and catalyze normative shifts pertaining to bystanders [63,64]. Notably, the findings from this scoping review corroborate the favorable reception of bystander training led by POLs affiliated with Greek Life, underscoring the resonance of such initiatives within this demographic.

Further reinforcing these observations, a systematic review of the bystander intervention training literature emphasizes the compelling impact of real-life scenarios and informal settings on engaging participants [15]. Studies advocating for naturalistic environments underscore the efficacy of informal settings in mitigating participant discomfort and distractions [49,56,62] compared to more formal, large-audience settings [50]. Such insights align with the broader literature on intervention efficacy, emphasizing the significance of relatable and comprehensible content delivery [65].

As Mujal and colleagues affirm, incentive is a potent tool for enhancing participant engagement [15]. Moreover, stakeholder buy-in is a pivotal determinant of training success, with institutional support from key entities like the Greek Life Office and the Dean of Students proving instrumental in facilitating training implementation [29,49].

Darlington (2014) and Pinkerton (2011) identified that quality content was a strength of their studies [49,57]. Darlington (2014) highlighted that an important evidence-based aspect of their training focused on environmental/group change by teaching participants how to support survivors [49]. Additionally, participants in the Pinkerton (2011) study appreciated the digestibility of the content, stating that the training was “easy to understand” [57]. These findings are supported by the bystander intervention training literature, in which others have found that evidence-based information and ensuring that content is easily understood by participants are essential for effective bystander intervention training [66]. By applying pedagogical principles to improve engagement, interventions can be better tailored to meet the needs of diverse participant groups, thereby enhancing training effectiveness and facilitating positive behavior change.

Contradictory elements emerged regarding the audience’s gender composition. Darlington (2014) emphasized the importance of tailoring the audience to a single gender, noting its effectiveness for women but reduced significance for men; yet, this still proved more effective than mixed-sex groups [49]. Conversely, Pinkerton (2011) received feedback from male participants who perceived the training as sexist and felt unfairly blamed. This study also revealed a desire among participants for co-ed training [57]. However, such contradictions may stem from specific training delivery methods. Given the overall lack of depth of the research on group composition for violence prevention training, the further development and testing of interventions are warranted to address these discrepancies effectively.

Discrepancies also arose regarding modality. Some studies [51,61,62] perceived in-person training to be an important best practice, while Ortiz and Shafer (2018) underscored the value of online tactics in fostering discussions and engaging a broader student audience [61]. Additionally, Pinkerton (2011) highlighted that participants perceived long sessions as unnecessary, suggesting that shorter, more frequent sessions might be better received [57]. Conversely, Bluth (2014) found that a longer format exhibited higher construct validity and Childers (2011) reported similar results between single-session and multi-session trainings [29,48]. While a meta-analysis [67] favored a higher session frequency for effectiveness, the literature also supports the impact of single-session interventions on participants’ understanding of SA. Moreover, Mujal and colleagues (2021) echoed these findings, noting the efficacy of in-person and online training, albeit with a slight advantage observed for in-person sessions [15].

The presence of contradictions within the identified best practices and barriers, such as the audience gender composition, in-person versus online trainings, and variations in training length and frequency, underscores the intricate nature of training design for this demographic. A nuanced approach, considering the audience, context, and training objectives, is crucial. These contradictions highlight the evolving nature of the field, emphasizing the ongoing need for more research in this area. Future studies should aim to understand and reconcile these findings, refining strategies for sexual assault prevention among Greek-affiliated college students and contributing to safer campus environments.

Campus staff typically implement these trainings and may prioritize the implementation capacity and fidelity over the evaluation and dissemination of results. Of note, there is no requirement to evaluate bystander intervention trainings included in the Campus SaVE, and any publications produced are often performed in collaboration with faculty and students. The majority of the studies included in this review used quantitative study designs, and measured program efficacy with validated measures commonly found in the SV literature, such as the bystander efficacy scale and the Illinois Rape Myth scale. The data reported here provide some guidance for how other studies should evaluate bystander intervention trainings. Many universities implement programs without conducting an evaluation, but these evaluations are needed and there is an opportunity to do so. This scoping review can provide a starting point for others wishing to evaluate their program implementation efforts. 

As researchers and practitioners strive to enhance the effectiveness of these trainings, there is a clear need for a more open and collaborative approach to sharing training details, fostering a broader understanding of effective interventions for Greek-affiliated college students. Expanding the literature in this area will not only deepen our understanding of effective practices, but also address the scarcity of available strategies for supporting behavior change among this high-risk demographic. Additionally, continuous dialogue and collaboration between researchers, educators, and Greek organizations are essential for the development of effective interventions, especially given the policy changes (i.e., the rollback of Title IX guidelines) introduced by Betsy DeVos’ leadership in the Department of Education [43]. These changes have resulted in uncertainty and confusion for campus staff and administration, highlighting the need for policy surrounding high-risk subgroups, such as cis men, athletes, and students in Greek Life, whose buy-in is critical for successful bystander action and reductions in violence perpetration [68,69]. 

This area remains understudied, and thus, there are no validated best practices for the implementation of bystander programs, including those for Greek-affiliated students. For the purposes of this paper, we define best practices as implementation strategies that are noted by the authors (of included studies, in each paper) to be associated with positive findings or reasons for successful implementation. Future research should work to identify and validate the standardized best practices for effective implementation in this context. Addressing broader policy implications and adopting a collaborative approach could contribute to creating safer and more secure environments on college campuses, aligning with the concerns raised by the Obama Administration regarding the sexual assault epidemic on college campuses.

### Limitations

It is important to acknowledge a notable limitation of this scoping review. Over half of the sources included in this review were unpublished dissertations, and despite this, only a fraction provided comprehensive (k = 2) or partial (k = 2) information about their training content. This striking gap highlights a significant issue within academia—the limitations placed on authors when publishing their research (e.g., publication bias, page limitations) can hinder the dissemination of comprehensive training material [70]. This, in turn, acts as a barrier to the utilization and evolution of bystander intervention training, which can result in uneven implementation and issues with fidelity. Moreover, only 18 studies met the criteria for this scoping review, highlighting the pressing need for more publications and research on existing bystander intervention trainings implemented for Greek-affiliated college students. The studies that met the inclusion criteria had a relatively short duration (1 day to 27 weeks). Thus, most were unable to measure the intervention effects in the long term. Research has shown that changes in behaviors as a result of interventions, such as changes in the rates of violence perpetration or victimization, may last beyond 12 months [71]. In order to determine if bystander intervention trainings for Greek-affiliated students are effective, researchers should consider longitudinal designs with an adequate follow-up time to observe the intended effects.

## 5. Conclusions

In conclusion, this scoping review provides a thorough examination of bystander intervention trainings tailored to Greek-affiliated college students, shedding light on their relevance to this high-risk demographic. The identified best practices and barriers underscore the significance of integrating elements such as relatability, comfort, engagement, and modality to optimize training effectiveness. Moreover, the efficacy of bystander interventions facilitated by peer opinion leaders (POLs) is highlighted, alongside the positive reception of POL-led initiatives within Greek Life. Studies also emphasize the impact of real-life scenarios and informal settings in enhancing participant engagement, echoing the broader literature on intervention efficacy. However, contradictions regarding the audience’s gender composition and modality highlight the intricacies of training design for this demographic, warranting further research. Collaboration and communication among stakeholders are pivotal in advancing our understanding of effective interventions. By expanding research efforts, we can effectively address the scarcity of strategies available for promoting bystander action among Greek-affiliated college students, thereby contributing to safer campus environments.

## Figures and Tables

**Table 1:** Individual study interventions, population, setting, requirement and length.

Citation	Pub Type and Source	Design	Intervention	Population and Setting	Sample Size and Age	Requirement	Length
Bluth, 2014 [29]	Dissertation—ProQuest LLC	Single group—no control group	Bringing in the Bystander	Sorority members at a midsize university in the Southern US	*n* = 13; 18 to 22	No	1.5 h
Cambron, 2014 [47]	Dissertation—ProQuest LLC	Quasi-experimental—with comparison group	Safe Sisters	Sorority members at University of West Florida (UWF)	*n* = 282; not available	No	1.5 h
Childers, 2011 [48]	Dissertation from ScholarWorks@UARK	Quasi-experimental—with comparison group	RESPECT	Undergraduate students at a Division I university in the Southern US	*n* = 100; 18 to 27	No	single class presentation
Darlington, 2014 [49]	Dissertation from ProQuest LLC	Randomized controlled trial—with control group	SWAT training (Sexual Wellness Advocacy Team) and SWAT Plus	Fraternity members at University of Oregon (UO), a large public university in the Pacific Northwest	*n* = 324; 18 to 25	No	SWAT 45 min; SWAT plus bystander 1.5 h
Davis, DeMaio & Fricker-Elhai, 2004 [50]	Journal Article from the Journal of Trauma Practice	Randomized controlled trial—with control group	Unspecified	Undergraduate women at 1 mid-sized public university and 2 southern universities	*n* = 310; 18 to 22	No	1 h
Devine, 2018 [51]	Thesis from ProQuest LLC	Quasi-experimental—no comparison group	Unspecified	Undergraduate students at a Midwestern university	*n* = 387; no available	No	Varied
Elias-Lambert & Black, 2016 [52]	Journal Article from the Journal of Interpersonal Violence	Quasi-experimental—with comparison group	Bringing in the Bystander	Undergraduate men at a large central university	*n* = 142; 18 to 26	Yes—fraternity members	1.5 h
Feldwisch et al., 2020 [53]	Journal Article from the Journal of College Counseling	Randomized controlled trial—with control group	Safe Sisters	Sorority members at a large midwestern university in the US	*n* = 139; 18 to 22	No	4 h
Foubert & Newberry, 2006 [54]	Journal Article from the Journal of College Student Development	Randomized controlled trial—with control group	The Men’s Program	Fraternity members at a small to midsized public institution in the south	*n* = 261; not available	No	1 h
Hahn et al., 2017 [55]	Journal Article from the Journal of Community Psychology	Quasi-experimental—no comparison group	The Women’s Program	Sorority women at a mid-sized university	*n* = 141; mean age of 19.49	No	1.5 h
Master et al., 2022 [56]	Journal Article from the Journal of American College Health	Quasi-experimental—no comparison group	Conversations and Pizza (CAP)	Fraternity members at an urban university in the Northeast of the US	*n* = 66; average age of 20	No	6 sessions over 3 months
Moynihan et al., 2011 [14]	Journal Article from Violence Against Women	Randomized controlled trial—with control group	Bringing in the Bystander	Sorority members and a northeastern public university	*n* = 56; average age of 19	No	1.5 h
Pinkerton, 2011 [57]	Dissertation from ProQuest LLC	Quasi-experimental—no comparison group	Men in Violence Prevention (MIVP)	Fraternity members at University of Hartford	*n* = 21; 18 to 19	Yes—new fraternity members	two 1-h sessions
Smith, 2013 [58]	Dissertation from ProQuest LLC	Quasi-experimental—with comparison group	Unspecified	Undergraduate students at a small independent liberal arts college in southern California	*n* = 1487; 18 to 24	Yes—orientation	55 min
Steward, 2018 [59]	Dissertation from ProQuest LLC	Quasi-experimental—with comparison group	Bringing in the Bystander and Sexpectations: Healthy relationships and sexuality for college students	Sorority members at the University of Tulsa	*n* = 102; average age of 19.6	Yes—sorority members	1.5 h
Taiwo, 2018 [60]	Conference Presentation from AcademyHealth	Quasi-experimental—no comparison group	Ten Man and Ten Woman Plan	Greek-affiliated undergraduate students at the University of Maryland	*n* = 109; not available	No	1 h sessions over nine weeks
Ortiz & Shafer, 2018 [61]	Conference Paper from the International Communication Association	Quasi-experimental—no comparison group	Unspecified	Undergraduate students at a large, public southwestern university	*n* = 992; mean age of 20.72	No	21 weeks
Brinkmeier, 2022 [62]	Dissertation from ProQuest LLC	Single group—no control group	Title IX Training	Fraternity members at 3 Midwestern universities (1 private and 2 public)	*n* = 28; not available	Varied	Varied

**Table 2:** Individual study designs and results.

Citation	Quality Appraisal	Measures	Time Period	Results
Bluth, 2014 [29]	63.9% (23/36)	Illinois Rape Myth Scale (IRMS); Readiness To Help Scale; Bystander Efficacy Scale; Attitudes Toward Women Scale (AWS)	pretest—(2 weeks)—pretest—intervention—posttest—(2 weeks)—posttest	Increase in bystander efficacy; Readiness to help remained the same; rape myth acceptance and AWS had a small positive change
Cambron, 2014 [47]	77.8% (28/36)	Decisional Balance Scale; Bystander Efficacy Scale; Perceived Threat Scale; Survivor Help Scale; Knowledge	Experimental: pretest—(1 week)—intervention—(1 weeks)—posttest Comparison: pretest—(1 week)—SV pamphlets—(1 weeks)—posttest	Significant increase in decisional balance, knowledge, and bystander efficacy for the treatment group; survivor help efficacy and perceived threat increased slightly
Childers, 2011 [48]	66.7% (24/36)	Bystander Efficacy Scale; Willingness to Engage in Bystander Behaviors; Readiness to Change	experimental: pretest—intervention—posttest—(1 month)—follow up comparison: posttest—(1 month)—follow up	Significant increase in bystander efficacy, willingness to engage in bystander behavior, and readiness to change which was maintained at 1 month follow-up; Gender, Greek affiliation, and athletic affiliation did not have an effect on these scores
Darlington, 2014 [49]	83.3% (30/36)	SV knowledge measure; Illinois Rape Myth Acceptance Scale; Sexual Consent Scale; Bystander Intention to Help Scale; Bystander Efficacy Scale; Decisional Balance Scale; Bystander Behavior Scale; Social Norms Measure; Sexual Experiences Survey; Social Desirability Inventory	experimental: pretest—intervention—posttest—(10–12 weeks)—follow up comparison: pretest—(2–4 weeks)—posttest—(10–12 weeks)—follow up	Interventions were effective at decreasing rape myth acceptance, knowledge of bystander behavior and bystander self-efficacy. SWAT plus was more effective at increasing bystander intervention behavior. SWAT was more effective at increasing the intention to help
Davis, DeMaio & Fricker-Elhai, 2004 [50]	77.8% (28/36)	Experiences with Violence Survey (EVS); Knowledge of Sexual Assault Survey (KSAS); Risk Questionnaire (RQ); Alcohol Consumption	experimental: pretest—intervention—posttest—(3 months)—follow up comparison: pretest—(3 months)—posttest—intervention—follow up	Intervention was effective in increasing knowledge of sexual assault issues; risky behaviors, substance use, and subsequent victimization were not reduced, regardless of victimization status
Devine, 2018 [51]	77.8% (28/36)	Bystander Efficacy Scale; two questions about previous engagement in bystander behavior	Posttest for all participants	Significant differences in self-efficacy between groups of students by type of bystander training; in-person training had a higher impact on self-efficacy; Greek affiliated students had high self-efficacy scores; participants were not more likely to intervene if they had high levels of self-efficacy; Greek affiliation was the only significant predictor of self-reported bystander intervention behaviors
Elias-Lambert & Black, 2016 [52]	80.6% (29/36)	Modified–Sexual Experiences Survey (M-SES); Marlowe–Crowne Social Desirability Scale–Short Form (MCSDS-SF); Illinois Rape Myth Acceptance Scale–Short Form (IRMAS-SF); Bystander Attitude Scale–Revised (BAS-R); Attraction to Sexual Aggression Scale–Modified (ASA); Bystander Behavior Scale–Revised (BBS-R)	experimental: pretest—intervention—posttest—(5 weeks)—follow up comparison: pretest—alternative treatment—posttest—(5 weeks)—follow up	Significant decrease in rape myth acceptance and sexually coercive behaviors overall; significant decrease in sexually coercive behavioral intentions but significantly rebounded at follow-up. Low-risk men showed positive change in rape myth acceptance and sexually coercive behavioral intention but high-risk men only showed significant change in sexual coercive behavior. No significant changes were found for either group for both bystander attitudes and bystander behaviors
Feldwisch et al., 2020 [53]	83.3% (30/36)	Readiness to Help; Bystander Efficacy Scale; Illinois Rape Myth Acceptance Scale; Intent to Help Scales	experimental: pretest—intervention—posttest control: pretest—(4 h)—posttest	Significant differences between treatment and waitlist control groups were shown on posttest scores for action, bystander efficacy, intent to help friends, and intent to help strangers. Significant differences were not shown between treatment and waitlist control groups on posttest scores for precontemplation, contemplation, and rape myth acceptance.
Foubert & Newberry, 2006 [54]	88.9% (32/36)	Illinois Rape Myth Acceptance Scale; Malamuth’s (1989) likelihood of raping scale; Rape Empathy Scale	G1: pretest—intervention (+alcohol module)—posttest G2: pretest—intervention (+alcohol and consent module)—posttest control: pretest	Those in the treatment groups had significant decreases in rape myth acceptance, likelihood of raping, and likelihood of committing sexual assault, and a significant increase in empathy toward survivors.
Hahn et al., 2017 [55]	77.8% (28/36)	Illinois Rape Myth Acceptance Scale; Sexual Assertiveness Scale; Bystander Efficacy Scale; Sexual Experiences Survey; Bystander Behavior Scale	pretest—intervention—(4 h)—posttest	Rape myth acceptance and bystander self-efficacy predicted bystander behaviors and sexual assertiveness; rape myth acceptance significantly deceased; no significant change in the bystander self-efficacy and sexual assertiveness
Master et al., 2022 [56]	72.2% (26/36)	Questions about consent, alcohol use, bystander intervention, sexual harass- ment, partner communication, contraception use, HIV testing, PrEP awareness and use, and HPV immunization history were adapted from previous studies	pretest—3 month long intervention—posttest	Increases in participants’ self-agency in the domains of personal responsibility; PrEP awareness increased greatly for participants (26% to 93%); HPV receipts increased from 60% to 93%; no change in the frequency of drinking alcohol or using drugs before having sex; no change in frequency of having sex or using contraception; increased recognition that sex while intoxicated is not harmless; significant findings regarding professed comfort/confidence in refusing sex if a partner was not wearing a condom and having conversations with partners about consent
Moynihan et al., 2011 [14]	80.6% (29/36)	Bystander Efficacy Scale; Bystander Intention to Help Scale; Readiness to Change Scale	Experimental: pretest—intervention—(5 weeks)—posttest Control: pretest—(5 weeks)—posttest	Intervention group showed improved efficacy or confidence about being a prosocial bystander, increased intention to help as bystanders, and greater sense of responsibility after attending the program compared to the control. Compared with the control group, the program participants showed significant intensity of change in a positive direction for bystander efficacy and responsibility and no significant backlash on any of the four outcome variables. No difference in the groups regarding denial that sexual and intimate partner violence are problems on campus. Scores for denial moved in the desired direction for both groups.
Pinkerton, 2011 [57]	75% (27/36)	Consumer Satisfaction Questionnaire; Reaction to Offensive Language and Behavior Scale; Rape Empathy Scale; Illinois Rape Myth Acceptance Scale	Pretest—intervention—posttest	Responses from the Elaboration Likelihood Model Questionnaire-Likert (ELMQ-L) portion of the consumer satisfaction questionnaire were quite favorable overall (84%). Results showed significant differences between pre-test scores and posttest scores for the Reaction to Offensive Language and Behavior (ROLB) subscales. No significant differences were observed between pre-test and posttest scores on measures assessing rape empathy or rape myth acceptance.
Smith, 2013 [58]	91.7% (33/36)	A twenty-four question survey instrument to assess student behavior was developed by the researcher	experimental: pretest—intervention—(6 weeks)—posttest comparison: pretest—(6 weeks)—posttest	The model was found to have significant effects, the most salient being that students who received the intervention made less risky decisions than those who did not attend the program. This change was observed in all three behavior domains. Special attention was paid to athletes, members of fraternities and sororities, and LGBT students. For Greek students who received the intervention, alcohol risk decreased significantly. For athletes who received the intervention, scores decreased significantly in regard to alcohol use as it relates to consent. Multiple linear regressions revealed that the intervention was a strong predictor of engaging in less risky behaviors around the use of alcohol and as it relates to consent.
Steward, 2018 [59]	88.9% (32/36)	Bystander Behavior Scale; Bystander Efficacy Scale; Bystander Intention to Help Scale; Sex-Related Alcohol Expectancy Scale; Illinois Rape Myth Acceptance Scale; Multidimensional Measure of Emotional Abuse; Alcohol Use Disorders Identification Test	G1: pretest—intervention 1—(1 month)—mid-assessment + intervention 2—(1 month)—posttest G2: pretest—intervention 1—(2 months)—posttest G3: pretest—intervention 2—(2 months)—posttest G4: pretest—(2 months)—posttest	No significant differences between the dependent variables for any of the four conditions. The only exception for the programming conditions was a significant increase in the bystander intention to help strangers and attitudes towards healthy relationships. For the comparison group, there were significant decreases in bystander behaviors and efficacy.
Taiwo, 2018 [60]	52.8% (19/36)	Bystander intention to help; rape myth acceptance	Pretest—9 week long intervention—posttest	Significant increases in bystander intention to help (BITH) scores from the pretest to the posttest. There were significant decreases in the rape myth acceptance (RMA) scores from pretest to posttest. RMA posttest scores for men were significantly higher than pretest scores. RMA posttest scores for women were significantly higher than pretest scores. For men, BITH posttest scores were marginally higher than pretest scores. For women, posttest bystander intention to help scores were not significantly higher than pretest scores.
Ortiz & Shafer, 2018 [61]	66.7% (24/36)	Sexual Consent Scale; Sexual Consent-Related Behavior Intentions Scale; in house measure of Accurate identification of sexual assault	Pretest—intervention—(6 weeks)—posttest—(21 weeks)—follow up	Students reported significantly greater positive attitudes about establishing sexual consent before engaging in sexual activity, preventing the use of behavioral control to obtain sexual consent from a partner, intentions to ask for consent prior to sexual activity and stopping sexual activity if consent is rescinded or if a partner is silent, and understanding what constitutes sexual consent (and sexual assault). While effective for the student population overall, further analyses revealed that members of social sororities and fraternities improved on all outcomes at a greater pace than non-members, and males improved at a greater pace than females on attitudes, intentions, and understanding of “clear” sexual assault situations. Finding that populations who were lower at baseline improved at a greater pace and also that all populations improved is encouraging.
Brinkmeier, 2022 [62]	94.4% (34/36)	Semi-structured interviews were conducted to answer the research questions	One interview per participant	The findings uncovered insights that would be useful to training efforts. For example, behaviors towards women are intimately tied to the foundation of fraternity affiliation—that is, relationships among members. Training is effective when it capitalizes on the value of relationships among brothers and women in sororities, as the self-image of fraternities has a significant influence on discourse and behaviors. The treatment of women can be leveraged by highlighting respect as a hallmark and indicator of fraternal reputation.

**Table 3:** Bystander programs, best practices and barriers.

Citation	Intervention	Best Practice	Barriers
Bluth, 2014 [29]	Bringing in the Bystander	Monetary incentive and interest in subject matter	Buy-in from campus stakeholders (Greek Life Office or Dean of Students; chapter participation incentives toward institutional mandates; competing programing; short sessions; only one session)
Cambron, 2014 [47]	Safe Sisters	Do not use scare tactics, instead focus on helping behaviors and bystander action	Not discussed
Childers, 2011 [48]	RESPECT	Peer leaders; single session	Not discussed
Darlington, 2014 [49]	SWAT training (Sexual Wellness Advocacy Team) and SWAT Plus	Naturalistic setting; evidence-based practices; peer facilitators; interactive programming; single-gender audiences; a focus on environmental or group change; Greek collaboration in program development; stakeholder buy-in	Facilitators not being Greek, 1 session
Davis, DeMaio & Fricker-Elhai, 2004 [50]	Unspecified	Not discussed	Large audience (more distractions, less private); didactic—little participant involvement
Devine, 2018 [51]	Unspecified	In person	Not discussed
Elias-Lambert & Black, 2016 [52]	Bringing in the Bystander	Not discussed	Not discussed
Feldwisch et al., 2020 [53]	Safe Sisters	Not discussed	Not discussed
Foubert & Newberry, 2006 [54]	The Men’s Program	Not discussed	Not discussed
Hahn et al., 2017 [55]	The Women’s Program	Not discussed	Not discussed
Master et al., 2022 [56]	Conversations and Pizza (CAP)	Informal structure	Not discussed
Moynihan et al., 2011 [14]	Bringing in the Bystander	Not discussed	Not discussed
Pinkerton, 2011 [57]	Men in Violence Prevention (MIVP)	Videos; engaging speakers; real life scenarios; good information; easily understood; judgement free zone; different perspectives, multi session	Longer sessions; male focus made participants feel attacked
Smith, 2013 [58]	Unspecified	Requirement for new students	Not discussed
Steward, 2018 [59]	Bringing in the Bystander and Sexpectations: Healthy relationships and sexuality for college students	Not discussed	Not discussed
Taiwo, 2018 [60]	Ten Man and Ten Woman Plan	Not discussed	Not discussed
Ortiz & Shafer, 2018 [61]	Unspecified	Variety of students in planning and implementation; sexual consent discussed by peers; interactive messaging; online and in-person training	Not discussed
Brinkmeier, 2022 [62]	Title IX Training	Partner with sorority for training; survivor stories; in-person training; interactive; peer led (other fraternity member or respected male involved in Greek life); informal	Not discussed

## Data Availability

Not applicable.
